# Mini-Review: Genetic Literacy and Engagement With Genetic Testing for Autism Spectrum Disorder

**DOI:** 10.3389/fgene.2021.693158

**Published:** 2021-06-29

**Authors:** India D. Little, Chris Gunter

**Affiliations:** ^1^Engagement Methods Unit, Social and Behavioral Research Branch, National Human Genome Research Institute, National Institutes of Health, Bethesda, MD, United States; ^2^Office of the Director, National Human Genome Research Institute, National Institutes of Health, Bethesda, MD, United States

**Keywords:** genetic literacy, genetic testing, autism spectrum disorder, neurodevelopmental assessment, science communication

## Abstract

As genomic and personalized medicine is integrated into healthcare, the need for patients to understand and make decisions about their own genetic makeup increases. Genetic literacy, or one’s knowledge of genetic principles and their applications, measures an individual’s ability to apply genetic information to their own treatment. Increased genetic literacy can improve comprehension of genetic tests and therefore increase participation in testing to detect and treat genetic disorders. It can also help providers understand and explain genetic information to their patients. However, current research indicates that the population’s genetic literacy is generally low. Because many medical students, providers, and patients cannot adequately apply genetic information to their health, new and beneficial genetic technologies can be underused. More specifically, though genetic testing is recommended at the time of diagnosis for those affected by autism spectrum disorder (ASD), as few as 22% of families undergo genetic testing after diagnosis. While ASD, a neurodevelopmental condition characterized by impaired social communication and restricted interests, has both genetic and environmental risk, genetic testing can give clinicians useful information and help families avoid potentially painful and costly tests, even when many families do not receive a “positive” genetic result through microarrays or gene panels. Improving genetic literacy in populations affected by ASD can also improve attitudes toward genetic testing, thereby ensuring access to genetic health risk information. In this mini review, we discuss the current literature describing genetic literacy and genetic testing rates for ASD.

## Introduction

Since the Human Genome Project completed in 2003, the use of genetic information in healthcare, as well as everyday life, has increased exponentially. In fact, leaders at the National Human Genome Research Institute predict that within the next decade, genetic testing will become a mainstream in healthcare, potentially becoming as commonplace as a complete blood count test (Green et al., 2020). There are increasingly more job opportunities in genetics, ancestry testing and clinical genetic testing is widely available, and individuals are able to participate in many facets of genetic research (Roberts M. C. et al., 2019). Celebrities have even publicized their genetic health decisions, drastically increasing awareness and interest in preventative genetic testing (Abrams et al., 2016). To prevent misconceptions regarding genetic risk, it is pivotal for the public to be equipped with accurate information and sufficient skills to make decisions about their own health and genomic data.

As genetic research expands, there is little doubt that our genes contribute to a variety of common and rare conditions (Claussnitzer et al., 2020). With the goal of prevention and treatment, genetic testing is often recommended as a way for clinicians to quantify and assess their patients’ disease risk. Genetic testing can provide information contributing to prevention and treatment for complex conditions, even though it is not always definitive. Receiving genetic risk information confirming a diagnosis can be comforting for patients and can even contribute to more healthful behaviors (McBride et al., 2010). A 2019 survey of two large research cohort studies in the US found that most participants had positive opinions of genetic testing, with a correlation between more favorable opinions and greater genomics knowledge or personal experience with genetic testing (Saylor et al., 2019). In clinical situations, many patients are not aware of the option for genetic testing or its benefits. The public must often rely on healthcare professionals to educate them and explain sometimes complex genetic results.

For those without a background in biology, understanding interactions between human health and chromosomal variants can be confusing and overwhelming. At a national level in the US, public understanding of how genetic information contributes to disease risk is generally low. In a survey distributed to 5,404 participants with secondary education, only 1.2% of the sample answered all of the basic genetic knowledge questions correctly (Chapman et al., 2019). Another national survey conducted in 2017 indicated that only half of individuals are aware of genetic testing and approximately a third were aware that genetic testing can contribute to disease treatment (Krakow et al., 2018). Similar trends are seen in healthcare education, with only 29% of a sample of 10,303 physicians reporting they received education in pharmacogenetic testing and only 25% of high school teachers reporting teaching contemporary issues in genetics (Kampourakis, 2016; Sabatello et al., 2019). Given the low rates of genetic knowledge in both the general public and providers, there is an ongoing effort by many organizations to both assess current knowledge rates and work to improve them (Green et al., 2020).

### Measuring Genetic Literacy

Genetic literacy, defined as “sufficient knowledge and understanding of genetic principles to make decisions that sustain personal well-being and effective participation in social decisions on genetic issues,” is one tool used to measure this phenomenon (Abrams et al., 2015). Importantly, genetic literacy is not the same as genetic knowledge. Those with high genetic literacy are able to understand their genetic testing results, communicate with their providers about genetic testing options, and make decisions about gene-related disease risk (Kampourakis, 2016). In research, genetic literacy has been defined and operationalized in many different ways. It has been measured using a person’s pronunciation of medical jargon or their knowledge of genes and heredity (Abrams et al., 2015). Because multiple measures for genetic literacy have been developed and optimized for various situations, it is difficult to adequately assess the public’s current genetic literacy rates and factors that influence it (Milo Rasouly et al., 2020). Abrams et al. (2015) proposed a measure of genetic literacy in three domains: Awareness knowledge, how-to knowledge, and principles knowledge. In conjunction, these domains assess the extent to which individuals are familiar with genetics concepts, their ability to apply genetic information to a particular health condition, and their factual genetic knowledge.

The same group assessed this measure in a nationally representative sample, applying genetic literacy to Angelina Jolie’s decision to pursue a prophylactic mastectomy following genetic testing in the *BRCA1/2* genes (Abrams et al., 2016). The results indicated moderate genetic knowledge, with the sample answering an average of half of the six factual genetics questions correctly. They also found an interesting interaction between confidence in one’s genetic knowledge, media exposure to Jolie’s decision, and genetic literacy. Those with high exposure to the news surrounding Jolie’s decision felt more confident about their genetic knowledge and their ability to apply this knowledge to the decision for surgery, regardless of their genetic literacy scores. Though it is beneficial for patients to feel confident in their health decisions, high-profile media can skew opinions about genetic health without a factual basis. For example, after the US Food and Drug Administration authorized a direct-to-consumer genetic test for three pathogenic variants in *BRCA1/2* in 2018, thousands of twitter messages relayed either information or opinion on the decision, with the most read being from established media outlets. Tweets from those expressing opinions most often focused on the harms of direct-to-consumer testing, without specifically referencing any research into the nature or frequency of these claims (Roberts M. C. et al., 2019). As discussions and media surrounding genetic health increase, it is important to counter false beliefs with accurate, research-based information. Patients and families with rare genetic diseases, for example, report that they have used social media to find each other and locate or vet potential treatments as they are developed (Iyer et al., 2020), a process which is fraught with the risk of misinformation that could misguide them.

### Population Differences in Genetic Literacy

Internationally, genetic knowledge and literacy rates vary as well. A large survey of willingness to share one’s genomic data, reporting on 36,268 individuals in 22 countries (Middleton et al., 2020), reported that “only 35.8% of the total sample say that they have some familiarity with the concepts” of DNA, genetics, and genomics; genetic literacy beyond that was not measured. Within the United States, over 30% of the 2,093-person sample indicated that they were unfamiliar with genetic concepts, while approximately 20% indicated they had personal experience with genetics, such as being a patient with a genetic condition or a genetics professional. This level of personal experience with genetics is relatively high; less than 12% of participants in Japan, Germany, Russia, and Mexico indicated familiarity through personal experience (Middleton et al., 2020). Higher levels in the US could be correlated with the increased use of direct-to-consumer genetic testing, emphasizing the need for genetic education as testing results become integrated into healthcare (Roberts J. et al., 2019).

Though many developed countries indicate relatively high awareness of genetics concepts, most individuals overestimate the impact of our genes on health (Kampourakis, 2016). The most common misconceptions state that genetic testing can control health outcomes, or that it exclusively determines your risk for a condition (Kampourakis, 2016). This is understandable in part because the most sophisticated research in most cases still does not adequately understand the interaction between genes and environment (Green et al., 2020); therefore, many in the public perhaps unsurprisingly attribute overall health to genetics exclusively and believe genetic traits to be immutable (Dar-Nimrod and Heine, 2011; Kampourakis, 2016). However, the belief that genetic information alone determines human traits or separates humans into strict groups ignores the social and environmental impact on human life and behavior. Unfortunately, and importantly, the perpetuation of this belief has led directly to discrimination between social groups when they are seen as genetically distinct and separate (Knerr et al., 2010). For example, genetic researchers in 2005 asserted that mutations in genes related to more adaptive brain development occurred more often in Eurasian than African populations. By suggesting that genes related to brain development are significantly different between ancestral groups, the researchers supported speculation that intelligence can vary by race (Knerr et al., 2010). Though the results were widely criticized within the field, the media only further emphasized the idea of strict and essential differences by genetic ancestry. Improving genetic literacy rates can diminish this perceived difference, educating individuals on the interaction between environment and genetics, and refuting the belief that genes are deterministic (Dar-Nimrod and Heine, 2011).

Genetic literacy rates also vary by social factors, including race, ethnicity, and socioeconomic status. Racial and ethnic minorities are less aware of genetic testing for cancer risk and are less likely to undergo such testing (Krakow et al., 2018). Additionally, individuals who are older or have lower incomes generally have lower genetic literacy and are even less likely to be aware of genetic tests (Krakow et al., 2018). Because those with low genetic literacy are less likely to participate in genetic research, they are less likely to benefit from scientific advances, such as genetic testing (Chapman et al., 2019). A previous study found that individuals undergoing genetic screening who showed low genetic literacy (independent of low genetic knowledge) were more likely to believe misconceptions about genomic medicine and less satisfied with the informed consent process for genetic research (Milo Rasouly et al., 2020). Such disparities in genetic literacy and awareness of genetic testing perpetuate existing health inequities in underserved populations. Without appropriate risk information, these populations are less likely to receive preventative information and adequate treatment.

Given that improving genetic literacy both increases awareness of genetic testing and improves attitudes toward genetic testing and its contributions to research, promoting genetic literacy and genetic testing awareness continues to be a public health goal for large organizations such as the National Human Genome Research Institute (Green et al., 2020).

### Genetic Testing for Autism Spectrum Disorder

We suggest that autism spectrum disorder (ASD) is a particular clinical example in which improving genetic literacy is important. Because the diagnosis process can be lengthy and grueling, children are often not diagnosed until years after displaying symptoms, which can impact functioning later in life. As detailed below, genetic testing can be useful in diagnosing ASD by shortening and improving the diagnostic process. However, the uptake of genetic testing in ASD is much lower than it could be, likely due to many factors including insufficient genetic literacy on all sides.

ASD is a neurodevelopmental condition characterized by restricted and repetitive interests as well as impairments in socialization and communication. ASD’s etiology is complex as it is influenced by a mix of genetic, epigenetic, and environmental factors. In the US, approximately 1 in 54 children reach the threshold for an ASD diagnosis and the average age of diagnosis is 4.25 years of age (Maenner et al., 2020). The potentially lengthy diagnostic process, often involving developmental pediatricians, neurologists, and geneticists, could mean a child is not diagnosed with ASD for years following initial symptoms. The age at which parents notice symptoms in their children depends on their awareness of ASD; first-time parents who are less aware of typical developmental milestones are less likely to notice developmental delays (Malik-Soni et al., 2021). Caregivers who notice symptoms in a child by 18 months of age are more likely to receive a prompt diagnosis, though many do not seek assessment until 35 months of age (Becerra-Culqui et al., 2018). In a large sample of families in the US and France, parents reported a significant delay between identifying symptoms at 29 months of age and receiving a diagnosis at approximately 55 months of age (Amiet et al., 2014). This gap represents a critical window of opportunity in which the child is missing out on support that can impact their functioning later in life (Li et al., 2016). Given the demonstration that some early behavioral interventions can change the trajectory of ASD (Siller, 2021) and the high frequency of co-occurring conditions which may need separate treatments, expediting the diagnostic process for ASD is imperative.

Because there is a clear genetic link to ASD, genetic testing is recommended by both the American College of Medical Genetic and Genomics and the American Academy of Pediatrics following an ASD diagnosis (Savatt and Myers, 2021). ASD is highly heritable, with estimates of twin heritability ranging from 70 to 90%, and recent advances in genetic research have identified over 100 gene or genetic variants associated with risk for ASD (Johannessen et al., 2016; Genovese and Butler, 2020; Satterstrom et al., 2020; Savatt and Myers, 2021). As with many other conditions that have a genetic basis, ASD genetic testing can provide families with an expedited and clearer diagnosis, giving them access to appropriate educational or therapy services. Because genetic testing is also used to determine the condition’s etiology, it can help children and families avoid other expensive or painful diagnostic tests, such as extensive neuroimaging, and metabolic testing including unnecessary blood draws. Conclusive results from genetic tests can also provide comfort to families affected by ASD. They can ease anxiety and uncertainty, aid medical and legal planning, and even provide a sense of empowerment and reduced negative emotions for the parents (Savatt and Myers, 2021).

Though genetic testing can provide many psychosocial benefits to families, it does not always yield a conclusive result. The first-tier test for ASD, chromosomal microarray or CMA, yields a diagnostic result in only 15–20% of cases (Savatt and Myers, 2021); in addition, the high frequency of copy number variants associated with ASD risk can produce test results which are not straightforward to interpret. The next logical test option is whole exome sequencing, which can increase the diagnostic yield up to 36% for neurodevelopmental disorders overall, making it the preferred genetic test for many clinicians (Srivastava et al., 2019; Martinez-Granero et al., 2021). Even though genetic tests can only provide a diagnostic result in some cases, they can help caregivers and providers identify areas of need and support in the child. In a survey of families who received CMA for ASD, over 60% of families reported that the testing was moderately to very helpful to the child and family (Reiff et al., 2015).

### Family Interest in and Referral Rates for ASD Genetic Testing

In a large Turkish sample, 87% of parents stated that they would pursue genetic testing if it could help identify the cause of their child’s ASD, and 84% believed that genetic testing referral is a key step in the diagnostic process (Ayhan et al., 2020). However, despite interest in and clinical recommendations for genetic testing, only about 22–28% of families undergo genetic testing in the US (Amiet et al., 2014; Zhao et al., 2019). This unexplained gap in genetic testing uptake is influenced by many factors including the high cost of genetic testing, lack of medical insurance, and low population genetic literacy.

Despite guidelines recommending genetic testing following an ASD diagnosis, referral rates from medical and genetics professionals are low (Amiet et al., 2014; Zhao et al., 2019). Ideally, families who have received an ASD diagnosis would be offered genetic testing and then counseling to determine whether testing is appropriate. However, because families may be referred to multiple medical professionals throughout the assessment, such as a geneticist, pediatrician, neurologist or genetic counselor, there is not always a logical or simple referral method. Many physicians report that they lack the specialized knowledge required to screen and diagnosis children with ASD (Malik-Soni et al., 2021). Medical guidelines also present conflicting information about which provider should offer a referral for genetic testing and when (Barton et al., 2018). As such, providers are left unsure of the specific genetic tests offered to families, and many providers have not received adequate training in treating autistic children and/or are unaware that genetic testing is an option for ASD (Barton et al., 2018; Malik-Soni et al., 2021). For example, the “gold-standard” diagnostic testing is often done by qualified psychologists who are not working with a medical team. As a result, of the majority of parents expressing interest in genetic testing, 83% report that they were not offered a referral by their doctor (Li et al., 2016). Child and adolescent psychiatrists may be better placed to order genetic testing, but a 2021 US survey indicated that only 32.7% had ordered a genetic test in relation to ASD in the previous 12 months (Soda et al., 2021). A mediating factor between low uptake of genetic testing and parental interest and medical guidelines recommending it is likely to be low population genetic literacy, in both families/individuals and providers. Indeed, in the survey of child and adolescent psychiatrists, those who had requested genetic testing related to ASD reported higher self-rated knowledge of genetic testing and higher perceived utility of genetic testing than those who had not (Soda et al., 2021). While this is to be expected perhaps, we suggest that there is work to be done in, for example, addressing the 50% or more of doctors in this survey who did not order genetic testing related to ASD even though they self-reported “good” or “very good” on both knowledge of genetic testing guidelines in psychiatry and knowledge about how to integrate genetic testing into practice (Soda et al., 2021).

## Discussion and Future Directions

For individuals and families affected by ASD, high genetic literacy indicates that one understands the genetic and environmental risk factors for ASD and can use this information to determine whether to pursue genetic testing. Parents with a positive association with genetic research are also more likely to support ASD genetic testing for their child (Floyd and Xu, 2017). In addition, those with higher genetic literacy are more willing to apply this knowledge to personal health decisions, potentially lessening the burden of any genetically based disease (Chapman et al., 2019; Mboowa and Sserwadda, 2019). Movements to involve families living with ASD in genetic research are already addressing this goal. One example is the SPARK project, which aims to be “the largest genetic study of autism ever.” In addition to creating and providing educational resources, SPARK has established a database that connects autistic individuals to researchers with the goal of developing new supports and treatments (Feliciano et al., 2018). It is important to note that some autistic advocates do not trust genetic or genomic research and have concerns about potentially harmful uses of technologies in this area. Improving interaction between autistic individuals and genetic researchers both fosters a collaborative and trusting relationship between healthcare professionals and their patients and improves accuracy of genetic education, in turn leading to higher genetic literacy.

We thus believe that healthcare will be improved by future research investigating genetic literacy rates in multiple population samples, and suggest that ASD is an illustrative test case where more research would be beneficial. Sufficient research can be followed by development of targeted genetic education resources addressing populations with lower genetic literacy. Examples include explainer websites targeted to families looking for resources at the time of diagnosis, like https://www.autismspeaks.org/expert-opinion/should-i-or-we-have-genetic-testing-autism or https://www.spectrumnews.org/news/genetic-testing-autism-explained/, or animated explainer videos such as https://youtu.be/LGQUE8fTx_A. Because the current level of genetic literacy is not sufficient to ensure individuals are educated to make informed decisions about their genetic information and health, we also recommend further research investigating genetic literacy and its relationship to attitudes toward genetic testing. Understanding the barriers to genetic literacy and genetic testing will help ensure equitable access to these rapidly expanding genetic technologies.

## Author Contributions

IL wrote the first draft of the manuscript. CG wrote sections and edited the manuscript. Both authors edited, read, and approved the submitted version.

## Conflict of Interest

The authors declare that the research was conducted in the absence of any commercial or financial relationships that could be construed as a potential conflict of interest.

## References

[B1] Abrams L. R. Koehly L. M. Hooker G. W. Paquin R. S. Capella J. N. McBride C. M. (2016). Media exposure and genetic literacy skills to evaluate Angelina Jolie’s decision for prophylactic mastectomy. *Public Health Genom.* 19 282–289. 10.1159/000447944 27427958 PMC12356274

[B2] Abrams L. R. McBride C. M. Hooker G. W. Cappella J. N. Koehly L. M. (2015). The many facets of genetic literacy: assessing the scalability of multiple measures for broad use in survey research. *PLoS One* 10:e0141532. 10.1371/journal.pone.0141532 26510161 PMC4625002

[B3] Amiet C. Couchon E. Carr K. Carayol J. Cohen D. (2014). Are there cultural differences in parental interest in early diagnosis and genetic risk assessment for autism spectrum disorder? *Front. Pediatr.* 2:32. 10.3389/fped.2014.00032 24795872 PMC4006049

[B4] Ayhan A. B. Beyazıt U. Topuz Ş Tunay ÇZ. Abbas M. N. Yılmaz S. (2020). Autism spectrum disorder and genetic testing: parents’ attitudes-data from Turkish sample. *J. Autism Dev. Disord.* 10.1007/s10803-020-04798-5 33222045

[B5] Barton K. S. Tabor H. K. Starks H. Garrison N. A. Laurino M. Burke W. (2018). Pathways from autism spectrum disorder diagnosis to genetic testing. *Genet. Med.* 20 737–744. 10.1038/gim.2017.166 29048417 PMC5908763

[B6] Becerra-Culqui T. A. Lynch F. L. Owen-Smith A. A. Spitzer J. Croen L. A. (2018). Parental first concerns and timing of autism spectrum disorder diagnosis. *J. Autism Dev. Disord.* 48 3367–3376. 10.1007/s10803-018-3598-6 29754290 PMC10289826

[B7] Chapman R. Likhanov M. Selita F. Zakharov I. Smith-Woolley E. Kovas Y. (2019). New literacy challenge for the twenty-first century: genetic knowledge is poor even among well educated. *J. Commun. Genet.* 10 73–84. 10.1007/s12687-018-0363-7 29589204 PMC6325037

[B8] Claussnitzer M. Cho J. H. Collins R. Cox N. J. Dermitzakis E. T. Hurles M. E. (2020). A brief history of human disease genetics. *Nature* 577 179–189. 10.1038/s41586-019-1879-7 31915397 PMC7405896

[B9] Dar-Nimrod I. Heine S. J. (2011). Genetic essentialism: On the deceptive determinism of DNA. *Psychol. Bull.* 137 800–818. 10.1037/a0021860 21142350 PMC3394457

[B10] Feliciano P. Daniels A. M. Green Snyder L. A. Beaumont A. Camba A. Esler A. (2018). SPARK: a US cohort of 50,000 families to accelerate autism research. *Neuron* 97 488–493. 10.1016/j.neuron.2018.01.015 29420931 PMC7444276

[B11] Floyd A. E. Xu L. (2017). Can genetic research motivate parents to pursue genomic testing for children with autism spectrum disorder? *J. Autism* 4:4. 10.7243/2054-992X-4-4

[B12] Genovese A. Butler M. G. (2020). Clinical assessment, genetics, and treatment approaches in autism spectrum disorder (ASD). *Int. J. Mol. Sci.* 21 1–18. 10.3390/ijms21134726 32630718 PMC7369758

[B13] Green E. D. Gunter C. Biesecker L. G. Di Francesco V. Easter C. L. Feingold E. A. (2020). Strategic vision for improving human health at the forefront of genomics. *Nature* 586 683–692. 10.1038/s41586-020-2817-4 33116284 PMC7869889

[B14] Iyer A. A. Barzilay J. R. Tabor H. K. (2020). Patient and family social media use surrounding a novel treatment for a rare genetic disease: a qualitative interview study. *Genet. Med.* 22 1830–1837. 10.1038/s41436-020-0890-6 32601388

[B15] Johannessen J. Nærland T. Bloss C. Rietschel M. Strohmaier J. Gjevik E. (2016). Parents’ attitudes toward genetic research in autism spectrum disorder. *Psychiatr. Genet.* 26 74–80. 10.1097/YPG.0000000000000121 26867185

[B16] Kampourakis K. (2016). Public understanding of genetic testing and obstacles to genetics literacy. *Mol. Diagn.* 27 469–477. 10.1016/B978-0-12-802971-8.00027-4

[B17] Knerr S. Ramos E. Nowinski J. Dixon K. Bonham V. L. (2010). Human difference in the genomic era: facilitating a socially responsible dialogue. *BMC Med. Genom.* 3:20. 10.1186/1755-8794-3-20 20504336 PMC2888748

[B18] Krakow M. Ratcliff C. L. Hesse B. W. Greenberg-Worisek A. J. (2018). Assessing genetic literacy awareness and knowledge gaps in the US population: Results from the health information national trends survey. *Public Health Genom.* 20 343–348. 10.1159/000489117 29852491 PMC6095736

[B19] Li M. Amuta A. Xu L. Dhar S. U. Talwar D. Jung E. (2016). Autism genetic testing information needs among parents of affected children: a qualitative study. *Patient Educ. Couns.* 99 1011–1016. 10.1016/j.pec.2015.12.023 26847420

[B20] Maenner M. J. Shaw K. A. Baio J. Washington A. Patrick M. DiRienzo M. (2020). Prevalence of autism spectrum disorder among children aged 8 Years-Autism and developmental disabilities monitoring network. *MMWR Surveill. Summ.* 69 1–12. 10.15585/MMWR.SS6904A1 32214087 PMC7119644

[B21] Malik-Soni N. Shaker A. Luck H. Mullin A. E. Wiley R. E. Lewis M. E. S. (2021). Tackling healthcare access barriers for individuals with autism from diagnosis to adulthood. *Pediatr. Res.* 1–8. 10.1038/s41390-021-01465-y 33767375 PMC7993081

[B22] Martinez-Granero F. Blanco-Kelly F. Sanchez-Jimeno C. Avila-Fernandez A. Arteche A. Bustamante-Aragones A. (2021). Comparison of the diagnostic yield of aCGH and genome-wide sequencing across different neurodevelopmental disorders. *NPJ Genom. Med.* 6:25. 10.1038/s41525-021-00188-7 33767182 PMC7994713

[B23] Mboowa G. Sserwadda I. (2019). Role of genomics literacy in reducing the burden of common genetic diseases in Africa. *Mol. Genet. Genom. Med.* 7:e776. 10.1002/mgg3.776 31131548 PMC6625136

[B24] McBride C. M. Koehly L. M. Sanderson S. C. Kaphingst K. A. (2010). The behavioral response to personalized genetic information: will genetic risk profiles motivate individuals and families to choose more healthful behaviors? *Annu. Rev. Public Health* 31 89–103. 10.1146/annurev.publhealth.012809.103532 20070198

[B25] Middleton A. Milne R. Almarri M. A. Anwer S. Atutornu J. Baranova E. E. (2020). Global public perceptions of genomic data sharing: what shapes the willingness to donate DNA and health data? *Am. J. Hum. Genet.* 107 743–752. 10.1016/j.ajhg.2020.08.023 32946764 PMC7536612

[B26] Milo Rasouly H. Cuneo N. Marasa M. DeMaria N. Chatterjee D. Thompson J. J. (2020). GeneLiFT: a novel test to facilitate rapid screening of genetic literacy in a diverse population undergoing genetic testing. *J. Genet. Couns.* 30 742–754. 10.1002/jgc4.1364 33368851 PMC8246865

[B27] Reiff M. Giarelli E. Bernhardt B. A. Easley E. Spinner N. B. Sankar P. L. (2015). Parents’ perceptions of the usefulness of chromosomal microarray analysis for children with autism spectrum disorders. *J. Autism Dev. Disord.* 45 3262–3275. 10.1007/s10803-015-2489-3 26066358 PMC4573251

[B28] Roberts J. Archer L. DeWitt J. Middleton A. (2019). Popular culture and genetics; friend, foe or something more complex? *Eur. J. Med. Genet.* 62 368–375. 10.1016/j.ejmg.2018.12.005 30590173 PMC6626485

[B29] Roberts M. C. Allen C. G. Andersen B. L. (2019). The FDA authorization of direct-to-consumer genetic testing for three BRCA1/2 pathogenic variants: a twitter analysis of the public’s response. *JAMIA Open* 2 411–415. 10.1093/jamiaopen/ooz037 32025636 PMC6993995

[B30] Sabatello M. Chen Y. Sanderson S. C. Chung W. K. Appelbaum P. S. (2019). Increasing genomic literacy among adolescents. *Genet. Med.* 21 994–1000. 10.1038/s41436-018-0275-2 30214064 PMC6417977

[B31] Satterstrom F. K. Kosmicki J. A. Wang J. Breen M. S. De Rubeis S. An J. Y. (2020). Large-scale exome sequencing study implicates both developmental and functional changes in the neurobiology of autism. *Cell* 180 568–584.e23. 31981491 10.1016/j.cell.2019.12.036 PMC7250485

[B32] Savatt J. M. Myers S. M. (2021). Genetic testing in neurodevelopmental disorders. *Front. Pediatr.* 9:526779. 10.3389/fped.2021.526779 33681094 PMC7933797

[B33] Saylor K. W. Ekunwe L. Antoine-LaVigne D. Sellers D. E. McGraw S. Levy D. (2019). Attitudes toward genetics and genetic testing among participants in the Jackson and Framingham heart studies. *J. Empir. Res. Hum. Res. Ethics* 14 262–273. 10.1177/1556264619844851 31068049 PMC6565476

[B34] Siller M. (2021). Editorial: individualizing interventions for young children with autism: embracing the next generation of intervention research. *J. Am. Acad. Child Adolesc. Psychiatry* 60 680–682. 10.1016/j.jaac.2020.11.003 33189877

[B35] Soda T. Pereira S. Small B. J. Torgerson L. N. Munoz K. A. Austin J. (2021). Child and adolescent psychiatrists’ perceptions of utility and self-rated knowledge of genetic testing predict usage for autism spectrum disorder. *J. Am. Acad. Child Adolesc. Psychiatry* 60 657–660. 10.1016/j.jaac.2021.01.022 33609654 PMC8404367

[B36] Srivastava S. Love-Nichols J. A. Dies K. A. Ledbetter D. H. Martin C. L. Chung W. K. (2019). Meta-analysis and multidisciplinary consensus statement: exome sequencing is a first-tier clinical diagnostic test for individuals with neurodevelopmental disorders. *Genet. Med.* 21 2413–2421. 10.1038/s41436-019-0554-6 31182824 PMC6831729

[B37] Zhao S. Chen W.-J. Dhar S. U. Eble T. N. Kwok O.-M. Chen L.-S. (2019). Genetic testing experiences among parents of children with autism spectrum disorder in the United States. *J. Autism Dev. Disord.* 49 4821–4833. 10.1007/s10803-019-04200-z 31542846

